# Clinical Presentation, Etiology, and Outcomes of HIV-Associated Cardiomyopathy: A Systematic Review of Published Case Reports

**DOI:** 10.3390/v18050510

**Published:** 2026-04-29

**Authors:** Omar Hozayen, Joseph Hozayen, Benjamin J. Behers, Anas Abu Jad, Bashar Roumia, Matthew W. Miller, Christoph A. Stephenson-Moe, Nicolas Riveros, Manuel Rosario, Karen M. Hamad

**Affiliations:** Internal Medicine Residency, Sarasota Memorial Hospital, Florida State University, Sarasota, FL 34239, USA; yousef.hozayen@med.fsu.edu (J.H.); bjb14@med.fsu.edu (B.J.B.); anas.abujad@med.fsu.edu (A.A.J.); bashar.roumia@med.fsu.edu (B.R.); matthew-miller@smh.com (M.W.M.); cstephensonmoe@med.fsu.edu (C.A.S.-M.); nicolas.riveros@med.fsu.edu (N.R.); m.a.rosario-espinal@med.fsu.edu (M.R.); karen.hamad@med.fsu.edu (K.M.H.)

**Keywords:** HIV-associated cardiomyopathy, systematic review, heart failure, antiretroviral therapy, opportunistic infections, drug-induced cardiomyopathy, myocarditis, dilated cardiomyopathy, global health disparities

## Abstract

HIV-associated cardiomyopathy is a significant cause of morbidity and mortality among people living with HIV, contributing to heart failure, arrhythmia, and sudden cardiac death. Despite its clinical importance, its individual-patient clinical spectrum has not been systematically synthesized. We conducted a systematic review of published English-language case reports and small case series describing cardiomyopathy in HIV-infected individuals. Etiologies were classified using a framework distinguishing cardiomyopathy arising from uncontrolled HIV from that occurring despite virologic control. Stratified analyses examined temporal trends and geographic differences. We identified 99 patients (75 male, 20 female, 4 unspecified) from 27 countries (80% high-income). Median age was 35 years (IQR 28–45). Among 52 patients with CD4 data, median was 154 cells/µL (IQR 84–391); 52% had CD4 < 200. Systolic dysfunction was present in 94% with echocardiographic data. Uncontrolled HIV phenotypes predominated (64%), but controlled phenotypes (21%)—including drug-induced cardiomyopathy (*n* = 19, predominantly zidovudine-associated) and autoimmune or inflammatory mechanisms (*n* = 13)—were substantial. Mortality declined across eras: 65% pre-ART, 32% early ART, 21% modern ART. Recovery occurred in 58%. HIV-associated cardiomyopathy is heterogeneous with improving outcomes across treatment eras. Systematic etiologic evaluation is warranted in all affected patients. The near absence of data from sub-Saharan Africa represents a critical gap.

## 1. Introduction

Among people living with HIV, the transition from acute infection to chronic disease with widespread antiretroviral therapy (ART) has shifted the burden of mortality toward non-infectious complications, with cardiovascular disease now representing a leading cause of death [1,2,3]. With widespread ART availability, HIV has transitioned from an acutely fatal infection to a chronic condition; nonetheless, individuals with HIV experience approximately 50% higher rates of cardiovascular events compared with uninfected controls, even after adjustment for traditional risk factors [4,5]. Among cardiac manifestations, cardiomyopathy remains particularly consequential, contributing to heart failure, arrhythmia, and sudden cardiac death [6]. A recent meta-analysis of 54 studies involving more than 125,000 adults with HIV reported pooled prevalences of 12% for left ventricular systolic dysfunction and 12% for dilated cardiomyopathy, with markedly higher rates in sub-Saharan Africa [7].

The SMART trial demonstrated a substantially increased risk of cardiovascular events—including a 57% higher rate—in patients assigned to CD4-guided ART interruption rather than continuous therapy [8,9]. The D:A:D study further identified associations between specific antiretroviral agents and myocardial infarction risk, including a 26% relative increase per year of cumulative ART exposure [10,11]. Screening echocardiography studies have estimated the prevalence of subclinical myocardial dysfunction at 10–50% across different populations [7,12]. However, cohort studies rarely include biopsy, pathogen-directed diagnostics, or advanced imaging, limiting mechanistic insight into the underlying causes of myocardial disease.

Case reports provide a unique and complementary source of information, often including detailed clinical reasoning, imaging, and histopathology. Lumsden and Bloomfield’s 2016 conceptual model distinguished cardiomyopathy occurring in uncontrolled HIV from that occurring despite virologic control—a framework summarized in Table 1 [13]. This distinction has informed contemporary thinking but has not been systematically evaluated using the cumulative published case literature.

A substantial knowledge gap persists: no prior study has systematically collected, standardized, and analyzed all published case reports of HIVAC. Because case reports often contain diagnostic detail unavailable in cohort datasets—including endomyocardial biopsy findings, cardiac MRI patterns, and pathogen-specific testing—they represent an underutilized resource for clarifying the etiologic spectrum of HIVAC.

We conducted the first systematic review of published HIVAC case reports, with objectives to characterize clinical presentation and outcomes at the individual patient level, classify etiologies according to the Tale of Two Worlds framework, examine temporal trends across treatment eras, and compare findings between high-income and low-/middle-income settings.

## 2. Methods

### 2.1. Search Strategy and Selection Criteria

We included case reports and small case series (≤10 patients with individual-level data) describing cardiomyopathy in individuals with confirmed HIV infection, provided clinical, imaging, or pathologic evidence of myocardial dysfunction was present, and sufficient detail was available to characterize presentation, etiology, or outcome. We excluded studies describing isolated diastolic dysfunction, pericardial disease without myocardial involvement, or structural heart disease unrelated to HIV. Cohort studies, cross-sectional studies, case–control studies, clinical trials, review articles, editorials, meta-analyses, and non-English publications were also excluded.

For the purposes of this review, cardiomyopathy was defined in accordance with established criteria as myocardial structural or functional disease—including dilated cardiomyopathy, myocarditis, or reduced left ventricular ejection fraction—not explained by coronary artery disease, hypertension, valvular disease, or congenital heart disease [14].

We systematically searched PubMed, Embase, and Cochrane CENTRAL from database inception through 30 October 2025. The PubMed search strategy was (HIV[tiab] OR HIV[mh] OR “human immunodeficiency virus”[tiab] OR AIDS[tiab] OR AIDS[mh] OR “acquired immunodeficiency syndrome”[tiab]) AND (cardiomyopathy[tiab] OR cardiomyopathy[mh] OR myocarditis[tiab] OR myocarditis[mh] OR “heart failure”[tiab] OR “heart failure”[mh] OR “ventricular dysfunction”[tiab] OR “dilated cardiomyopathy”[tiab] OR “cardiac dysfunction”[tiab] OR “left ventricular dysfunction”[tiab]) AND (“case reports”[pt] OR “case report”[tiab] OR “case series”[tiab] OR “case study”[tiab]). The Embase search strategy was (hiv:ti OR ‘human immunodeficiency virus’:ti OR ‘hiv-1’:ti OR ‘hiv 1’:ti OR ‘aids’:ti) AND (cardiomyopathy:ti,ab OR myocarditis:ti,ab OR ‘dilated cardiomyopathy’:ti,ab OR myopericarditis:ti,ab OR ‘left ventricular dysfunction’:ti,ab) AND ‘case report’. The Cochrane CENTRAL search strategy was (HIV OR “human immunodeficiency virus” OR AIDS) AND (cardiomyopathy OR myocarditis OR “heart failure” OR “ventricular dysfunction”). Reference lists of included studies were hand-searched to identify additional cases. This review was conducted and reported in accordance with PRISMA guidelines [15]. The protocol was prospectively registered with PROSPERO (CRD420251181209).

### 2.2. Data Collection Process

Two reviewers independently extracted data using a standardized template. Variables included demographics, HIV disease parameters (CD4 count, viral load, ART status), presenting symptoms, laboratory and electrocardiographic findings, echocardiographic and cardiac MRI parameters, endomyocardial biopsy results, author-stated etiologies, treatments, and clinical outcomes. Discrepancies were resolved by consensus. Each case report was treated as a unique case. Included cases were cross-referenced by age, sex, country, and year of publication to assess for duplicate patient reporting across studies.

### 2.3. Data Items

Etiologies were classified according to a structured framework adapted from Lumsden and Bloomfield’s “Tale of Two Worlds” model [13], distinguishing mechanisms associated with uncontrolled HIV infection from those occurring despite virologic control. Each case was assigned a single dominant etiology, with adjudication guided by a prespecified hierarchy in which opportunistic infection myocarditis superseded tuberculous involvement, both of which superseded direct HIV myocarditis; drug-induced and autoimmune or immune-mediated mechanisms were considered only when higher-tier causes were absent, and micronutrient deficiency was assigned solely when no alternative explanation was evident. When more than one mechanism was described, this hierarchy was applied to identify the most specific and proximate cause. Mixed-phenotype cases—defined as those with two or more explicitly documented mechanisms—were counted descriptively but retained a single assignment for analysis. Cases were designated unclassified when the available clinical, laboratory, imaging, and pathologic data did not support assignment to any etiologic category.

### 2.4. Stratified Analyses

We performed prespecified stratified analyses by publication era (pre-ART [before 1996], early ART [1996–2006], and modern ART [2007 and later]) and by country income level (high-income [HIC] vs. low-/middle-income [LMIC] per World Bank classification). These stratifications were chosen to examine whether etiologic patterns and outcomes have evolved with changing treatment paradigms and to assess potential differences between resource settings.

### 2.5. Statistical Analysis

Continuous variables are presented as means with interquartile ranges (IQRs) or medians with IQRs where appropriate. Categorical data are presented as counts and percentages with denominators reflecting available data for each variable. All analyses were performed using Microsoft Excel. No statistical software package was used given the descriptive nature of this review. No inferential statistics were performed. Because this review is based on case reports and small case series, the data are not suitable for estimating prevalence or incidence of HIV-associated cardiomyopathy. Denominators reflect the number of patients with available data for each variable and therefore vary across outcomes.

### 2.6. Role of the Funding Source

There was no funding source for this study.

## 3. Results

### 3.1. Study Selection

**Figure 1:**
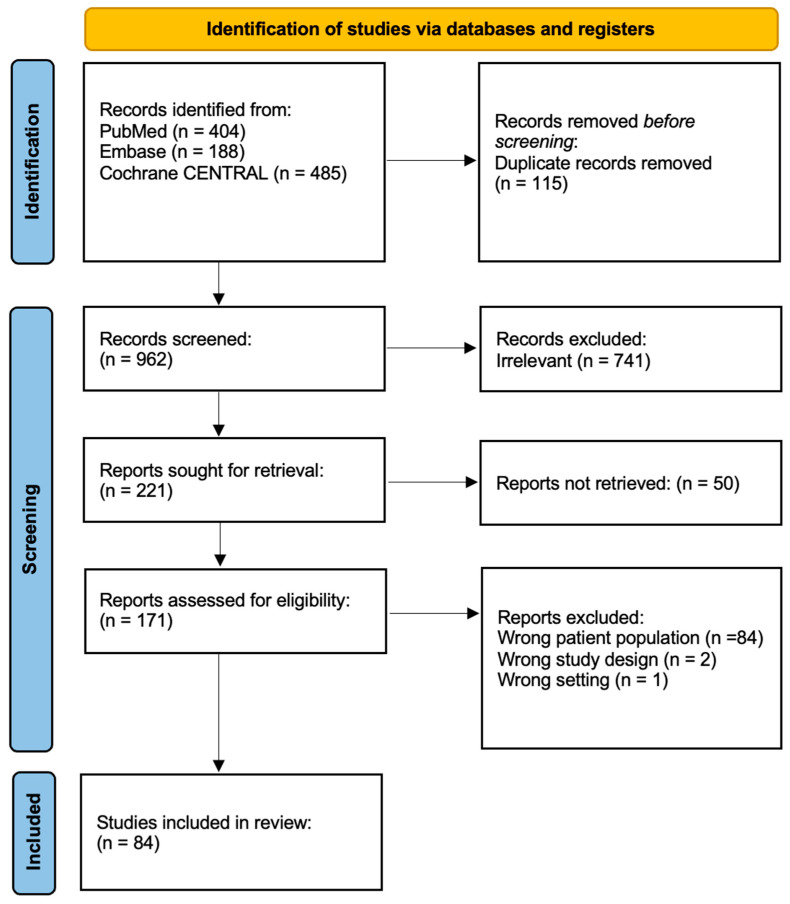
PRISMA flow diagram demonstrating the process of data collection.

As shown in Figure 1, from 1077 identified records (PubMed: 404; Embase: 188; Cochrane CENTRAL: 485), 115 duplicates were removed. Screening of the remaining 962 records led to the exclusion of 741 irrelevant studies. Of the 221 reports sought for retrieval, 171 were successfully obtained and assessed for eligibility. After exclusion of 87 reports for wrong population, design, or setting, 84 case reports meeting all eligibility criteria were included, comprising 99 unique individual patients across 27 countries [16,17,18,19,20,21,22,23,24,25,26,27,28,29,30,31,32,33,34,35,36,37,38,39,40,41,42,43,44,45,46,47,48,49,50,51,52,53,54,55,56,57,58,59,60,61,62,63,64,65,66,67,68,69,70,71,72,73,74,75,76,77,78,79,80,81,82,83,84,85,86,87,88,89,90,91,92,93,94,95,96,97,98,99].

### 3.2. Patient Characteristics

**Figure 2:**
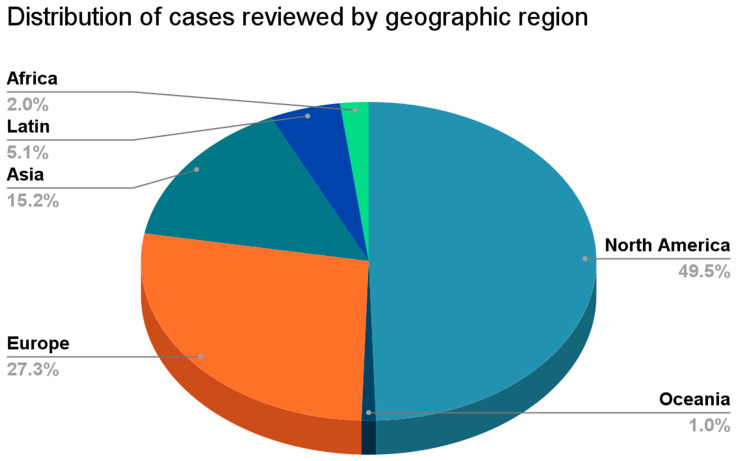
Geographic Distribution of Included Cases.

The United States contributed the largest number of cases (45), followed by the United Kingdom (7), Italy (5), Spain (4), India (4), and Germany (3). The remaining 29 cases originated from 21 additional countries across Europe, Asia, Latin America, Africa, and Oceania. The geographic distribution of cases is shown in Figure 2. Overall, 80% of cases originated from high-income countries and 17% from low- and middle-income countries (Table 2).

Median age was 35 years (IQR 28–45; range 0.16–78). The cohort included 75 males (76%), 20 females (20%), and 4 patients with sex unspecified. Twenty-six patients (26%) presented with cardiomyopathy at the time of initial HIV diagnosis.

CD4 count at presentation was reported in 52 patients (53%). Among these, median CD4 was 154 cells/µL (IQR 84–391). Approximately half of cases with reported CD4 count at presentation had advanced immunosuppression: 27/52 (52%) with CD4 < 200 cells/µL, 17/52 (33%) had a CD4 count within 200–500, and 8/52 (15%) had CD4 > 500 (32).

Viral load was reported in 36 patients (36%): 27 (75%) had detectable viremia and 9 (25%) had undetectable or suppressed viral load (<200 copies/mL). Fifty-nine patients (60%) were not on ART at presentation, while 39 (39%) were receiving ART (Table 3).

Data completeness across key clinical, laboratory, and outcome variables is summarized in Table 4.

### 3.3. Presenting Features

Dyspnea was the predominant presenting symptom (70/99, 71%), followed by peripheral edema (40%), fatigue (37%), fever (35%), cough (23%), palpitations (21%), chest pain (20%), orthopnea (8%), and syncope (6%) (Table 5).

### 3.4. Imaging and Pathology

Troponin was elevated in 21/35 patients tested (60%); in 65 patients’ troponin was not reported, reflecting the historical timeframe of many cases. BNP was elevated in 11/13 patients tested (85%). ECG abnormalities were common: T-wave or ST-T changes (26%), sinus tachycardia (25%), ST-elevation (6%), atrial fibrillation (4%), and complete heart block (2%). Only 3% had documented normal ECG.

Among 64 patients with quantifiable echocardiographic data, 60 (94%) demonstrated reduced LV ejection fraction and 4 (6%) had preserved ejection fraction. Cardiac MRI was performed in 14 of 99 patients (14%); among those imaged, myocarditis-pattern findings were observed in 10 (71%), while 2 (14%) had normal studies and 2 had other or nonspecific abnormalities. Coronary artery disease was definitively excluded in 38 of 99 patients (38%) through coronary angiography or CT angiography.

Endomyocardial biopsy was performed in 22 patients (22%). Biopsy demonstrated lymphocytic myocarditis in 13 patients (59%), interstitial fibrosis in 2 (9%), normal myocardial architecture in 1 (5%), and nonspecific or mixed inflammatory changes in 5 (23%).

Autopsy-derived myocardial pathology was available in 27 patients (27%). Pathogen-confirmed opportunistic infection myocarditis was identified in 14 (52%), including Toxoplasma gondii, cytomegalovirus, and cryptococcal organisms. Inflammatory infiltrates were present in 4 cases (15%), myocarditis was explicitly described in 2 (7%), and features of chronic structural remodeling—dilated cardiomyopathy, myocyte hypertrophy, or interstitial fibrosis—were documented in 4 (15%), 6 (22%), and 5 (19%) patients, respectively. Two autopsies (7%) demonstrated minimal or normal myocardial findings, and one (4%) was limited by autolysis. Cardiac imaging and pathology findings are summarized in Table 6.

**Table 6:** Cardiac Imaging and Pathology.

Modality	Finding	*n*/N (%)
Echocardiography	Reduced LVEF	60/64 (94%)
	Preserved LVEF	4/64 (6%)
Cardiac MRI	Performed	14/99 (14%)
	Myocarditis pattern	10/14 (71%)
	Normal MRI	2/14 (14%)
	Other/nonspecific	2/14 (14%)
Endomyocardial biopsy	Performed	22/99 (22%)
	Myocarditis	13/22 (59%)
	Fibrosis	2/22 (9%)
	Normal	1/22 (5%)
	Nonspecific or mixed non-inflammatory changes	5/22 (23%)
Autopsy pathology	Performed	27/99 (27%)
	Opportunistic infection myocarditis (pathogen-confirmed)	14/27 (52%)
	Inflammatory infiltrates	2/27 (7%)
	Myocarditis (explicit)	5/27 (19%)
	Dilated cardiomyopathy	2/27 (7%)
	Myocyte hypertrophy	6/27 (22%)
	Fibrosis	4/27 (15%)
	Normal/minimally abnormal	4/27 (15%)
	Autolysis/non-diagnostic	1/27 (4%)

### 3.5. Etiologic Categories and Phenotypes

Applying the Tale of Two Worlds framework, all 99 cases were assessed for etiologic classification (Table 7). Uncontrolled HIV phenotypes predominated (64%), with direct HIV myocarditis/toxicity most common (42 cases), followed by opportunistic infection (23 cases—including toxoplasmosis, CMV, cryptococcus, and EBV), micronutrient deficiency (3 cases), and tuberculosis (1 case).

Controlled HIV phenotypes accounted for 21%. Drug-induced cardiomyopathy was identified in 19 cases. Among these, Zidovudine (AZT) was implicated in at least 10 cases, Didanosine (ddI) in 4, and Dolutegravir in 1 case. Autoimmune or inflammatory mechanisms were identified in 13 cases. Nine cases had features of both phenotypic categories and were assigned to the uncontrolled phenotype per the prespecified hierarchy. Thirteen cases (13%) remained unclassified.

### 3.6. Treatment and Outcomes

In-hospital outcome data were available for 94 patients. Thirty-five died (37%) and 59 survived (63%). Recovery or improvement in cardiac function was documented in 56/96 (58%).

In-hospital mortality across eras was 65% (17/26) in the pre-ART era, 32% (7/22) in the early ART era, and 21% (9/43) in the modern ART era (Table 8). Recovery rates were 46% (13/28), 70% (14/20), and 64% (28/44), respectively.

Seventy-nine cases originated from high-income countries and seventeen from low-/middle-income countries (Table 9). Crude mortality appeared higher in HIC cases (31/74, 42%) than in LMIC cases (4/17, 24%), but this difference was largely confounded by era: all pre-ART cases were reported exclusively from HIC settings, whereas LMIC cases were published only during the ART era. When restricted to the modern ART era, mortality was similar (HIC: 6/28 [21%] vs. LMIC: 3/12 [25%]). Recovery rates were modestly higher in LMIC cases (12/17, 71%) compared with HIC cases (43/76, 57%). Access to advanced diagnostics differed substantially: endomyocardial biopsy was performed in 22/79 HIC cases (28%) and in 0/17 LMIC cases.

In-hospital therapeutic strategies reflected a combination of guideline-directed heart failure therapy, antiretroviral optimization, and etiology-specific treatment. Loop diuretics (91%), ACE inhibitors or ARBs (80%), and beta-blockers (74%) were the most frequently used heart-failure medications. ART was initiated in 86% of ART-naïve patients and modified in 50% of those already receiving therapy. Etiology-specific antimicrobial therapy—targeting toxoplasmosis (7/7), tuberculosis (8/8), CMV (2/2), and parasitic infection (1/1)—was consistently applied when pathogens were identified. Corticosteroids were administered in 15% of cases and IVIG in 6%. Advanced mechanical circulatory support was uncommon (ECMO 3%; temporary MCS 5%). One patient underwent orthotopic heart transplantation, representing the most aggressive form of rescue therapy used in the cohort. Details of in-hospital therapeutic strategies are summarized in Table 10.

**Table 10:** In Hospital Therapy.

Therapy Category	Treatment	*n*/N (%)
Heart-failure therapy	Loop diuretics	85/93 (91%)
	ACE inhibitor or ARB	72/90 (80%)
	Beta-blocker	68/92 (74%)
	Aldosterone antagonist	41/87 (47%)
	ARNI (sacubitril–valsartan)	3/99 (3%)
	Ivabradine	2/99 (2%)
	Digoxin	7/92 (8%)
Antiretroviral therapy (ART)	ART initiation (ART-naïve patients)	48/56 (86%)
	ART modification (patients already on ART)	18/36 (50%)
Etiology-specific therapy	Anti-toxoplasma therapy	7/7 (100%)
	Anti-TB therapy	8/8 (100%)
	CMV-directed therapy	2/2 (100%)
	Albendazole (parasitic)	1/1 (100%)
Immunomodulatory therapy	Corticosteroids	15/99 (15%)
	IVIG	6/99 (6%)
	Other immunosuppression	2/99 (2%)
Advanced cardiac support	ECMO	3/99 (3%)
	Temporary mechanical circulatory support (IABP/Impella)	5/99 (5%)
	Permanent device (ICD/CRT)	2/99 (2%)
Transplantation	Orthotopic heart transplantation	1/99 (1%)

## 4. Discussion

### 4.1. Principal Findings

This systematic review provides the first individual-patient-level synthesis of HIV-associated cardiomyopathy (HIVAC) and characterizes its global clinical and etiologic landscape. These observations reflect patterns within published cases and should not be interpreted as estimates of population-level disease burden. From 99 cases originating across 27 countries, several important observations emerge.

The most notable finding is the substantial improvement in clinical outcomes over time. Crude in-hospital mortality fell from 65% in the pre-ART era to 21% in the modern ART era, representing more than a three-fold reduction. Recovery rates improved in parallel: overall, 56% of patients with reported follow-up demonstrated meaningful recovery of left ventricular function. These trends likely reflect not only advances in HIV management but also heightened recognition of HIVAC, more consistent use of echocardiography and cardiac MRI, and greater availability of etiology-specific therapy. The high recovery rate emphasizes that HIVAC is frequently reversible when appropriately diagnosed and treated.

Our mortality estimates are considerably higher than those reported in contemporary cohort studies. For example, the HIV-HEART study observed a 5-year mortality of approximately 8% among HIV-positive individuals with cardiac dysfunction, and the SMART and D:A:D cohorts reported similarly low cardiovascular mortality. Case reports preferentially describe severe, unusual, or fatal presentations, and the overrepresentation of pre-ART cases magnifies this effect. Accordingly, the rates reported here should be interpreted as reflecting the severe end of the HIVAC severity spectrum.

### 4.2. Etiologic Phenotypes

Drug-induced cardiomyopathy emerged as a meaningful contributor, identified in 19 cases (≈19%). Zidovudine accounted for at least half of these, consistent with the recognized mitochondrial toxicity of early nucleoside analogue reverse-transcriptase inhibitors. This is consistent with observational data: the D:A:D cohort documented a progressive increase in cardiovascular risk with cumulative antiretroviral exposure [10,11], and echocardiographic screening studies have estimated the prevalence of subclinical myocardial dysfunction at 10–50% among ART-treated patients [7,12]. Although zidovudine use has markedly declined, the finding underscores the necessity of reviewing antiretroviral therapy in all patients with unexplained cardiomyopathy. Several additional cases demonstrated autoimmune or inflammatory mechanisms within controlled HIV phenotypes, highlighting the evolving spectrum of HIVAC in the modern ART era.

### 4.3. Geographic and Temporal Patterns

Global disparities in published cases were marked. While 80% of cases originated from high-income countries (HICs) and 17% from low-/middle-income countries (LMICs; 3% unclassified), this likely reflects differences in research infrastructure, access to advanced diagnostics, and English-language publication norms rather than a true difference in disease burden. Diagnostic resources differed sharply: endomyocardial biopsy was performed in 28% of HIC cases but in 0% of LMIC cases. Because HIV burden is highest in sub-Saharan Africa and South Asia—settings where advanced cardiac imaging and biopsy are rarely available—these findings likely reflect a profound reporting gap. This is supported by Erqou et al., whose meta-analysis of over 125,000 adults with HIV found a higher prevalence of LVSD in African studies (17.2%) compared with North American and European studies (13.8%), and specifically noted higher DCM prevalence in the African region [7], suggesting that LMIC populations bear a disproportionate burden of HIVAC that is poorly captured in the published case literature. The absence of biopsy and limited access to cardiac MRI in these regions further restrict the ability to confirm myocarditis, identify opportunistic infections, or detect ART-related mechanisms.

## 5. Limitations

This review has several limitations. As a retrospective synthesis of published case reports, causal inference is inherently limited. Observed distributions of etiologies and outcomes reflect reporting patterns rather than true epidemiologic frequencies, as case reports disproportionately represent rare, dramatic, or fatal presentations, inflating mortality estimates. Etiologic assignments rely on authors’ interpretations, which may reflect incomplete diagnostics or local resource constraints; notably, the near-complete absence of biopsy and advanced cardiac imaging in LMIC settings limits the validity of direct etiologic comparisons across resource settings. Survival bias likely exists, as patients dying before diagnostic evaluation are less likely to be reported. The restriction to English-language publications may exclude relevant cases from high-burden non-English-speaking regions. Temporal heterogeneity was substantial, with cases spanning more than three decades during which HIV epidemiology, treatment paradigms, and diagnostic tools changed dramatically. Additionally, our etiologic classification framework, while prespecified, represents one of several possible approaches, and alternative hierarchies could yield modestly different distributions. Information on traditional cardiovascular risk factors (e.g., hypertension, diabetes, smoking) was inconsistently reported, limiting assessment of their contribution to cardiomyopathy risk and outcomes. Finally, missing data were common: CD4 counts were available in only 53% of patients and viral load in 36%, limiting correlation of disease control with phenotype or outcome.

## 6. Clinical Implications

HIV-associated cardiomyopathy should be approached as a heterogeneous and potentially reversible condition requiring systematic etiologic evaluation. Clinicians should consider opportunistic infections, antiretroviral drug toxicity, and immune-mediated mechanisms in all affected patients. Early diagnostic workup—including echocardiography, cardiac MRI where available, and pathogen-directed testing—is supported by the high observed rate of cardiac recovery with appropriate therapy. Prospective studies from high-burden LMIC regions are needed.

## 7. Conclusions

This systematic review synthesizes individual-patient data from 99 published cases of HIV-associated cardiomyopathy across 27 countries and nearly four decades. Outcomes reported in case reports appear to have improved across treatment eras, although these estimates are subject to publication bias and should not be extrapolated to broader HIV populations. While uncontrolled HIV phenotypes predominate, controlled phenotypes—particularly drug-induced cardiomyopathy—may be more frequently reported than traditionally recognized. The near absence of published cases from sub-Saharan Africa likely reflects a reporting gap rather than true differences in disease burden and highlights an important priority for future prospective research. These findings support consideration of systematic etiologic evaluation, with attention to both infectious and non-infectious mechanisms, in patients presenting with HIV-associated cardiomyopathy.

## Figures and Tables

**Table 1:** Etiologies and Characteristic Phenotypes of HIV-Associated Cardiomyopathy.

Disease State	Characteristic HIVAC Phenotype	Etiology of HIVAC
Uncontrolled HIV Disease (i) Immunosuppressed host (ii) High viral load (iii) Low CD4 count (<400 cells/mm^3^)	(i) Myocarditis (a) Direct HIV toxicity (b) Opportunistic Infections (1) Viral: Coxsackie B, CMV, EBV (2) Non-viral: Toxoplasmosis, Cryptococcus, MAC	(i) More commonly seen in LMIC (ii) Symptomatic, systolic dysfunction +/− dilated ventricles (iii) Poor prognosis
	(ii) Tuberculous Myopericarditis	
	(iii) Micronutrient Deficiency (a) Selenium Deficiency	
Controlled HIV Disease (i) Immunocompetent host (ii) Undetectable viral load	(i) Cardiac Autoimmunity	(i) More commonly seen in HIC (ii) Subclinical diastolic dysfunction with increased strain patterns
	(ii) Cardiac inflammation	
	(iii) ART toxicity (a) AZT-induced cardiomyopathy	

Adapted from Lumsden and Bloomfield, 2016.

**Table 2:** Patient Characteristics (N = 99).

Characteristic		Value
Age, median (IQR) in years		35 (28–45)
Sex	Male	75 (75%)
	Female	20 (20%)
	Not specified	4 (4%)
Geographic region	North America	49 (49%)
	Europe	27 (27%)
	Asia	15 (15%)
	Latin America/Caribbean	15 (15%)
	Africa	2 (2%)
	Oceania	1 (1%)
Income setting	High-income country	79 (80%)
	Low-/middle-income country	17 (17%)
Publication era	Pre-ART (<1996)	29 (29%)
	Early ART (1996–2006)	21 (21%)
	Modern ART (2007+)	44 (44%)

**Table 3:** HIV Disease Parameters.

Parameter		*n*/N (%)
CD4 count (cells/µL), median (IQR)		154 (IQR 84–391), *n* = 52
	CD4 < 200	27/52 (52%)
	CD4 200–500	17/52 (36%)
	CD4 > 500	8/52 (15%)
Viral load reported		36/99 (36%)
	Detectable	27/36 (75%)
	Undetectable/suppressed	9/36 (25%)
On ART at presentation		39/99 (39%)
Not on ART at presentation		59/99 (60%)
Primary HIV diagnosis at presentation		26/99 (26%)

**Table 4:** Data Completeness of Key Clinical, Laboratory, and Outcome Variables.

Variable	Reported *n*/99 (%)	Missing *n*/99 (%)
Age	92 (93%)	7 (7%)
Sex	95 (96%)	4 (4%)
CD4 count	52 (53%)	47 (47%)
Viral load	36 (36%)	63 (64%)
ART status	98 (99%)	1 (1%)
Echocardiographic LVEF	64 (65%)	35 (35%)
Troponin	35 (35%)	64 (65%)
BNP	13 (13%)	86 (87%)
Survival outcome	94 (95%)	5 (5%)
Cardiac function recovery	96 (97%)	3 (3%)

**Table 5:** Clinical Presentation.

Symptom	*n*/99 (%)
Dyspnea	70 (71)
Peripheral edema	40 (40)
Fatigue	37 (37)
Fever	35 (35)
Cough	23 (23)
Palpitations	21 (21)
Chest pain	20 (20)
Orthopnea	8 (8)
Syncope	6 (6)

**Table 7:** Etiologic Classification.

Etiologic Category	Phenotypes	N (%)
Uncontrolled HIV phenotypes, 63 (64%)	Direct HIV myocarditis/uncontrolled HIV	42 (42%)
	Opportunistic infection myocarditis	23 (23%)
	Tuberculous myopericarditis	1 (1%)
	Micronutrient deficiency	3 (3%)
Controlled HIV phenotypes, 21 (21%)	Drug-induced cardiomyopathy	19 (19%)
	Autoimmune/inflammatory	13 (13%)
Unclassified, 15 (15%)		
Total, 99 (100%)		

Subcategory counts reflect all documented mechanisms and are not mutually exclusive. Nine cases had features of both phenotypic categories and were assigned to the uncontrolled phenotype per the prespecified hierarchy. Subcategory totals therefore exceed phenotype totals.

**Table 8:** Stratified Analysis by Publication Era.

Variable	Pre-ART (<1996) *n* = 29	Early ART (1996–2006) *n* = 22	Modern ART (2007+) *n* = 44
Mortality	17/26 (65%)	7/22 (32%)	9/43 (21%)
Recovery	13/28 (46%)	14/20 (70%)	28/44 (64%)

**Table 9:** Stratified Analysis by Income Setting.

Variable	HIC (*n* = 79)	LMIC (*n* = 17)
Era distribution		
Pre-ART (<1996)	29 (37%)	0 (0%)
Early ART (1996–2006)	18 (23%)	4 (24%)
Modern ART (2007+)	29 (37%)	12 (71%)
Mortality (crude)	31/74 (42%)	4/17 (24%)
Mortality (modern era only)	6/28 (21%)	3/12 (25%)
Recovery	43/76 (57%)	12/17 (71%)
Biopsy performed	22/79 (28%)	0/17 (0%)

## Data Availability

Data is available from the corresponding author upon reasonable request.

## References

[1] Feinstein M.J., Bahiru E., Achenbach C., Longenecker C., Hsue P., So-Armah K., Freiberg M., Lloyd-Jones D. (2016). Patterns of cardiovascular mortality for HIV-infected adults in the United States: 1999 to 2013. Am. J. Cardiol..

[2] Croxford S., Kitching A., Desai S., Kall M., Edelstein M., Skingsley A., Burns F., Copas A., Brown A., Sullivan K. (2017). Mortality and causes of death in people diagnosed with HIV in the era of highly active antiretroviral therapy compared with the general population. Lancet Public Health.

[3] Shah A.S.V., Stelzle D., Lee K.K., Beck E., Shirjel A., Clifford S., Longenecker C., Strachan F., Bagchi S., Whiteley W. (2018). Global burden of atherosclerotic cardiovascular disease in people living with HIV. Circulation.

[4] Freiberg M.S., Chang C.C., Kuller L.H., Skanderson M., Lowy E., Kraemer K.L., Butt A.A., Goetz M.B., Leaf D., Oursler K.A. (2013). HIV infection and the risk of acute myocardial infarction. JAMA Intern. Med..

[5] Freiberg M.S., Chang C.H., Skanderson M., Patterson  O.V., DuVall S.L., Brandt C.A., So-Armah K.A., Vasan  R.S., Oursler  K.A., Gottdiener J. (2017). Association between HIV infection and the risk of heart failure with reduced ejection fraction and preserved ejection fraction in the antiretroviral therapy era. JAMA Cardiol..

[6] Sinha A., Feinstein M.J. (2020). Epidemiology, pathophysiology, and prevention of heart failure in people with HIV. Prog. Cardiovasc. Dis..

[7] Erqou S., Lodebo B.T., Masri A., Altibi A.M., Echouffo-Tcheugui J.B., Dzudie A., Ataklte F., Choudhary G., Bloomfield G.S., Wu W.-C. (2019). Cardiac dysfunction among people living with HIV: A systematic review and meta-analysis. JACC Heart Fail..

[8] El-Sadr W.M., Lundgren J.D., Neaton J.D., Gordin F., Abrams D., Arduino R.C., Babiker A., Burman W., Clumeck N., Cohen C.J. (2006). CD4+ count-guided interruption of antiretroviral treatment. N. Engl. J. Med..

[9] Phillips A.N., Carr A., Neuhaus J., Visnegarwala F., Prineas R., Burman W.J., Williams I., Drummond F., Duprez D., Belloso W.H. (2008). Interruption of antiretroviral therapy and risk of cardiovascular disease in persons with HIV-1 infection: Exploratory analyses from the SMART trial. Antivir. Ther..

[10] Friis-Møller N., Sabin C.A., Weber R., d’Arminio Monforte A., El-Sadr W.M., Reiss P., Thiébaut R., Morfeldt L., De Wit S., Pradier C. (2003). Combination antiretroviral therapy and the risk of myocardial infarction. N. Engl. J. Med..

[11] Friis-Møller N., Reiss P., Sabin C.A., Weber R., d’Arminio Monforte A., El-Sadr W.M., Thiébaut R., De Wit S., Kirk O., Fontas E. (2007). Class of antiretroviral drugs and the risk of myocardial infarction. N. Engl. J. Med..

[12] Mondy K.E., Gottdiener J., Overton E.T., Henry K., Bush T., Conley L., Hammer J., Carpenter C.C., Kojic E., Patel P. (2011). High prevalence of echocardiographic abnormalities among HIV-infected persons in the era of highly active antiretroviral therapy. Clin. Infect. Dis..

[13] Lumsden R.H., Bloomfield G.S. (2016). The causes of HIV-associated cardiomyopathy: A tale of two worlds. Biomed Res. Int..

[14] Elliott P., Andersson B., Arbustini E., Bilinska Z., Cecchi F., Charron P., Dubourg O., Kühl U., Maisch B., Mckenna W.J. (2008). Classification of the cardiomyopathies: A position statement from the European Society of Cardiology Working Group on Myocardial and Pericardial Diseases. Eur. Heart J..

[15] Page M.J., McKenzie J.E., Bossuyt P.M., Boutron I., Hoffmann T.C., Mulrow C.D., Shamseer L., Tetzlaff J.M., Akl E.A., Brennan S.E. (2021). The PRISMA2020 statement: An updated guideline for reporting systematic reviews. BMJ.

[16] Steinherz L.J., Brochstein J.A., Robins J. (1986). Cardiac involvement in congenital acquired immunodeficiency syndrome. Am. J. Dis. Child..

[17] Calabrese L.H., Proffitt M.R., Yen-Lieberman B., Hobbs R.E., Ratliff N.B. (1987). Congestive cardiomyopathy and illness related to the acquired immunodeficiency syndrome (AIDS) associated with isolation of retrovirus from myocardium. Ann. Intern. Med..

[18] Lafont A., Wolff M., Marche C., Clair B., Regnier B. (1987). Overwhelming myocarditis due to Cryptococcus neoformans in an AIDS patient. Lancet.

[19] Brady M.T., Reiner C.B., Singley C., Roberts W.H., Sneddon J.M. (1988). Unexpected death in an infant with AIDS: Disseminated cytomegalovirus infection with pancarditis. Pediatr. Pathol..

[20] Levy W.S., Varghese P.J., Anderson D.W., Leiboff R.H., Orenstein J.M., Virmani R., Bloom S. (1988). Myocarditis diagnosed by endomyocardial biopsy in human immunodeficiency virus infection with cardiac dysfunction. Am. J. Cardiol..

[21] Mullins R.J., Bastian B., Sutherland D.C. (1988). AIDS and the heart. Aust. N. Z. J. Med..

[22] Adair O.V., Randive N., Krasnow N. (1989). Isolated toxoplasma myocarditis in acquired immune deficiency syndrome. Am. Heart J..

[23] Cregler L.L., Sosa I., Ducey S., Abbey L. (1990). Myopericarditis in acquired immunodeficiency syndrome diagnosed by gallium scintigraphy. J. Natl. Med. Assoc..

[24] Lipshultz S.E., Fox C.H., Perez-Atayde A.R., Sanders S.P., Colan S.D., McIntosh K., Winter H.S. (1990). Identification of human immunodeficiency virus-1 RNA and DNA in the heart of a child with cardiovascular abnormalities and congenital acquired immune deficiency syndrome. Am. J. Cardiol..

[25] Matturri L., Quattrone P., Varesi C., Rossi L. (1990). Cardiac toxoplasmosis in pathology of acquired immunodeficiency syndrome. Panminerva Med..

[26] Schindler J.M., Neftel K.A. (1990). Simultaneous primary infection with HIV and CMV leading to severe pancytopenia, hepatitis, nephritis, perimyocarditis, myositis, and alopecia totalis. Klin. Wochenschr..

[27] Hakas J.F., Generalovich T. (1991). Spontaneous regression of cardiomyopathy in a patient with the acquired immunodeficiency syndrome. Chest.

[28] Leidig G.A. (1991). Clinical, echocardiographic, and electrocardiographic resolution of HIV-related cardiomyopathy. Mil. Med..

[29] Maserati R., Parisi A., Pan A., Lanzarini L. (1991). Rapidly reversible cardiomyopathy in an AIDS patient. AIDS.

[30] Miller R.F., Gilson R., Hage C., Scaravilli F., Michaels L. (1991). HIV-associated dilated cardiomyopathy. Genitourin. Med..

[31] Cappell M.S., Mikhail N., Ortega A. (1992). Toxoplasma myocarditis in AIDS. Am. Heart J..

[32] Herskowitz A., Willoughby S.B., Baughman K.L., Schulman S.P., Bartlett J.D. (1992). Cardiomyopathy associated with antiretroviral therapy in patients with HIV infection: A report of six cases. Ann. Intern. Med..

[33] Balaz A. (1993). CMV myocarditis in a patient with HIV disease. AIDS Patient Care.

[34] Bruneel F., Gachot B., Lucet J.C., Bedos J.P., Wolff M. (1993). Shoshin beriberi in a patient with human immunodeficiency virus infection. Intensive Care Med..

[35] Albrecht H., Stellbrink H.J., Fenske S., Schafer H., Greten H. (1994). Successful treatment of Toxoplasma gondii myocarditis in an AIDS patient. Eur. J. Clin. Microbiol. Infect. Dis..

[36] Kovacs A., Hinton D.R., Wright D., Xu J., Li X.L., Rasheed S., Hofman F. (1996). Human immunodeficiency virus type 1 infection of the heart in three infants with acquired immunodeficiency syndrome and sudden death. Pediatr. Infect. Dis. J..

[37] Monsuez J.J., Ferchal F., Evans J., Lachurie M.L., Passeron J. (1996). Acute myocarditis mimicking myocardial infarction in an HIV infected patient. Eur. Heart J..

[38] Steinherz L.J. (1996). Cardiomyopathy related to acquired immunodeficiency syndrome in children. J. Pediatr..

[39] Fath-Ordoubadi F., van der Watt M.J., Noble M.I. (1997). Early presentation of dilated cardiomyopathy as a part of seroconversion illness in human immunodeficiency virus infection. Clin. Cardiol..

[40] Suarez Fernandez J., Merino Arribas J.M., Rodrigo Palacios J., Gonzalez Vilchez F. (1997). [Regressive dilated cardiac myopathy in a girl with HIV infection]. An. Esp. Pediatr..

[41] Tambini R., Fiocchi R., Gavazzeni G., Perani V., Mamprin F., Delvecchio G., Fracassetti O. (1997). Human immunodeficiency virus infection after heart transplantation: Development of cardiomyopathy after long-term survival. Int. J. Infect. Dis..

[42] Kent S., Ferguson M., Trotta R., Jordan L. (1998). T wave alternans associated with HIV cardiomyopathy, erythromycin therapy, and electrolyte disturbances. S. Med. J..

[43] Eerens F., Van Cleemput J., Peetermans W.E. (1999). A probable primary HIV infection associated with acute non-specific myocarditis causing severe dilated cardiomyopathy. Acta Clin. Belg..

[44] Fuster M., Negredo E., Cadafalch J., Domingo P., Illa I., Clave P. (1999). HIV-associated polymyositis with life-threatening myocardial and esophageal involvement. Arch. Intern. Med..

[45] Saulsbury F. (2001). Resolution of organ-specific complications of human immunodeficiency virus infection in children with use of highly active antiretroviral therapy. Clin. Infect. Dis..

[46] Frerichs F.C., Dingemans K.P., Brinkman K. (2002). Cardiomyopathy with mitochondrial damage associated with nucleoside reverse-transcriptase inhibitors. N. Engl. J. Med..

[47] Diogenes M.S., Carvalho A.C., Succi R.C. (2003). Reversible cardiomyopathy subsequent to perinatal infection with the human immunodeficiency virus. Cardiol. Young.

[48] Brucato A., Colombo T., Bonacina E., Orcese C., Vago L., Oliva F., Distefano G., Frigerio M., Paino R., Violin M. (2004). Fulminant myocarditis during HIV seroconversion: Recovery with temporary left ventricular mechanical assistance. Ital. Heart J..

[49] Srivastava M., Verghese C., Sepkowitz D. (2004). Acute reversible heart failure with highly active antiretroviral therapy. Am. J. Ther..

[50] Breuckmann F., Neumann T., Kondratieva J., Wieneke H., Ross B., Nassenstein K., Barkhausen J., Kreuter A., Brockmeyer N., Erbel R. (2005). Dilated cardiomyopathy in two adult HIV-positive patients possibly related to highly active antiretroviral therapy. Eur. J. Med. Res..

[51] Peter A.A., Seecheran S. (2005). Multiple ventricular thrombus in HIV cardiomyopathy. Heart.

[52] Eza D.E., Lucas S.B. (2006). Fulminant toxoplasmosis causing fatal pneumonitis and myocarditis. HIV Med..

[53] Lanjewar D.N., Agale S.V., Chitale A.R., Joshi S.R. (2006). Sudden death due to cardiac toxoplasmosis. J. Assoc. Physicians India.

[54] Sa I., Moco R., Cabral S., Reis A.H., Pereira L.S., Torres S., Gomes J.L. (2006). Constrictive pericarditis of tuberculous etiology in the HIV-positive patient: Case report and review of the literature. Rev. Port. Cardiol..

[55] Chimenti C., Del Nonno F., Topino S., Abbate I., Licci S., Paglia M.G., Capobianchi M.R., Petrosillo N., Frustaci A. (2007). Fatal myocardial co-infection by Toxoplasma gondii and Parvovirus B19 in an HIV patient. AIDS.

[56] Lopriore E., Rozendaal L., Gelinck L.B., Bokenkamp R., Boelen C.C., Walther F.J. (2007). Twins with cardiomyopathy and complete heart block born to an HIV-infected mother treated with HAART. AIDS.

[57] Oberdorfer P., Sittiwangkul R., Puthanakit T., Pongprot Y., Sirisanthana V. (2008). Dilated cardiomyopathy in three HIV-infected children after initiation of antiretroviral therapy. Pediatr. Int..

[58] Rogers J.S., Zakaria S., Thom K.A., Flammer K.M., Kanno M., Mehra M.R. (2008). Immune reconstitution inflammatory syndrome and human immunodeficiency virus-associated myocarditis. Mayo Clin. Proc..

[59] Syed F.F., Aje A., Ntsekhe M., Mayosi B.M., Moosa S., Tshifularo M., Smedema J.P. (2008). Resolution of nodular myocardial tuberculosis demonstrated by contrast-enhanced magnetic resonance imaging. Cardiovasc. J. Afr..

[60] McArthur M.A., Kalu S.U., Foulks A.R., Aly A.M., Jain S.K., Patel J.A. (2009). Twin preterm neonates with cardiac toxicity related to lopinavir/ritonavir therapy. Pediatr. Infect. Dis. J..

[61] Mehmood S., Blais D., Martin S., Sai-Sudhakar C. (2009). Heartmate XVE destination therapy for end-stage heart failure in a patient with human immunodeficiency virus. Interact. Cardiovasc. Thorac. Surg..

[62] Pano-Pardo J.R., Alcaide M.L., Abbo L., Dickinson G. (2009). Primary HIV infection with multisystemic presentation. Int. J. Infect. Dis..

[63] Aziz F., Doddi S., Penupolu S. (2010). Human immunodeficiency virus-associated myocarditis. Internet J. Intern. Med..

[64] Raj V., Joshi S., Pennell D.J. (2010). Cardiac magnetic resonance of acute myocarditis in an HIV patient presenting with acute chest pain syndrome. Circulation.

[65] Patane S., Marte F., Sturiale M., Dattilo G., Albanese A. (2011). Myocarditis and cardiomyopathy HIV associated. Int. J. Cardiol..

[66] Bal A., Dhooria S., Agarwal R., Garg M., Das A. (2014). Multiple and atypical opportunistic infections in a HIV patient with Toxoplasma myocarditis. Cardiovasc. Pathol..

[67] Frustaci A., Petrosillo N., Francone M., Verardo R., Ippolito G., Chimenti C. (2014). Biopsy-proven autoimmune myocarditis in HIV-associated dilated cardiomyopathy. BMC Infect. Dis..

[68] Tabata N., Yamamuro M., Sugiyama S., Mizobe M., Takashio S., Tsujita K., Yamamoto E., Tanaka T., Kojima S., Kaikita K. (2014). A case of human immunodeficiency virus-related heart failure resembling dilated cardiomyopathy but accompanied by high cardiac output. J. Cardiol. Cases.

[69] Thawabi M., Habib M., Shaaban H., Shamoon F. (2014). Acute eosinophilic myocarditis and hyper IgE in HIV infection: A case report. N. Am. J. Med. Sci..

[70] Aliku T.O., Lubega S., Lwabi P. (2015). Resolution of dilated cardiomyopathy in an adolescent with change of a failing highly active antiretroviral drug therapy. Afr. Health Sci..

[71] Kiselnik D., Wolak A., Abu-Shakra M., Basok A. (2015). Acute myocarditis and myopathy as presenting manifestations of human immunodeficiency virus infection. Isr. Med. Assoc. J..

[72] Nkoke C., Kuate L.M., Luchuo E.B., Edie S.D., Boombhi J., Menanga A. (2015). Biventricular thrombi in dilated cardiomyopathy in a patient with human immunodeficiency virus infection: A case report. BMC Res. Notes.

[73] Suwatcharangkoon S., Meads D.B., Tegeler C.H., Reynolds P.S. (2015). Transcranial Doppler sonography: Atypical dicrotic pulse waveforms in a man with HIV infection and severe cardiomyopathy. J. Neuroimaging.

[74] Cuervo G., Simonetti A.F., Alegre O., Sanchez-Salado J.C., Podzamczer D. (2016). Toxoplasma myocarditis: A rare but serious complication in an HIV-infected late presenter. AIDS.

[75] Mahlab-Guri K., Asher I., Rosenberg-Bezalel S., Elbirt D., Burke M., Sthoeger Z.M. (2016). Two case reports of severe myocarditis associated with the initiation of dolutegravir treatment in HIV patients. Medicine.

[76] Tariq S., Raza A., Narurkar R., Aronow W., Ahmed A., Panza J., Cooper H., Lanier G., Gass A., Aggarwal C. (2017). Combined heart-kidney transplantation in a patient with human immunodeficiency virus. J. Am. Coll. Cardiol..

[77] Vandi G., Calza L., Girometti N., Manfredi R., Musumeci G., Bon I., Re M.C. (2017). Acute onset myopericarditis as unusual presentation of primary HIV infection. Int. J. STD AIDS.

[78] Eyer-Silva W.A., Rosa da Silva G.A., da Cunha Pinto J.F. (2019). Acute myocarditis after switch to dolutegravir: A reminder of potential toxicity of integrase inhibitor-including HAART. AIDS.

[79] DeFilippis E.M., Kumbhakar R., Pereira M.R., Marboe C.C., Maurer M.S. (2020). Eosinophils, lymphocytes, and myocytes, oh my: HIV-associated myocarditis. Am. J. Med..

[80] Kammari C.B., Rallabandi S., Rallabandi H., Daggubati S.R., Adapa S., Naramala S., Konala V.M. (2020). Dilated cardiomyopathy with biventricular thrombus secondary to impaired coagulation in a patient with HIV. F1000Research.

[81] Kara M., Hancerli S., Bayramoglu Z., Kaba O., Aliyev B., Ozdemircioglu F., Haccacoglu E.E., Acunas B., Nisli K., Somer A. (2020). The report of cardiomyopathy and mid aortic syndrome in a HIV infected child. Malawi Med. J..

[82] Kutscher E., Kladney M. (2020). A case of new HIV presenting with cardiomyopathy and nephropathy. J. Gen. Intern. Med..

[83] Peters L.L., Ambardekar A.V. (2020). Successful heart transplantation after giant cell myocarditis in a patient with HIV complicated by recurrent giant cell myocarditis and Kaposi sarcoma. J. Heart Lung Transplant..

[84] Al-Maqbali J.S., Al-Sibani M., Al-Maqrashi N., Al Alawi A.M., Al Lawati H. (2021). Rivaroxaban for treatment of left ventricular thrombus: A case report. Am. J. Case Rep..

[85] Chango Azanza D.X., Fernandez B., Vasquez Ortiz Z., Chapa M., Rosales Uvera S. (2021). Cardiac multimodality imaging assessment of dystrophic myocardial calcification in a human immunodeficiency virus-infected patient with dilated cardiomyopathy. Cureus.

[86] Mastroianni A., Guadagnino G., Greco S., Urso F., Mauro M.V., Vangeli V. (2021). Efficacy of ivabradine in HIV-associated dilated cardiomyopathy. Recent. Prog. Med..

[87] Stobe S., Tayal B., Tunnemann-Tarr A., Hagendorff A. (2021). Dynamics in myocardial deformation as an indirect marker of myocardial involvement in acute myocarditis due to HIV infection: A case report. Eur. Heart J. Case Rep..

[88] Szakal-Toth Z., Szlavik J., Soltesz A., Berzsenyi V., Csikos G., Varga T., Racz K., Kiraly A., Sax B., Hartyanszky I. (2021). Acute heart transplantation from mechanical circulatory support in a human immunodeficiency virus-positive patient with fulminant myocarditis. ESC Heart Fail..

[89] Aquino-Bruno H., Andrade-Cuellar E.N., Morales-Portano J.D., Alcantara-Melendez M.A. (2023). Percutaneous mitral and tricuspid edge-to-edge repair as a bridge therapy to heart transplantation in advanced heart failure secondary to human immunodeficiency virus: A case report. Eur. Heart J. Case Rep..

[90] Mohan A., Reddy S., Mirza N., Suleiman A. (2023). Toxoplasma cardiomyopathy in a 36 year HIV positive female. Circulation.

[91] Togashi D., Harada T., Nakajima I., Kasagawa A., Nakayama Y., Sasaki K., Akashi Y.J. (2023). Successful epicardial radiofrequency ablation of ventricular tachycardia in a patient with human immunodeficiency virus-associated cardiomyopathy. Int. Heart J..

[92] Wicaksono A., Martini H., Rahimah A.F., Kurniawan A. (2023). A young male patient with cardiomyopathy associated with human immunodeficiency virus (HIV) infection in the era of highly active antiretroviral therapy. Indones. J. Cardiol..

[93] Kharel M., Subedi A., Hossain M.F. (2024). HIVAC (HIV-associated cardiomyopathy): An overlooked complication of HIV in developing countries. Clin. Case Rep..

[94] Sabri M., Janga C., Riaz F., Nugooru S., Haas D. (2024). Human immunodeficiency virus associated cardiomyopathy: A rare cause of heart failure with reduced ejection fraction in era of highly active antiretroviral therapy. Circulation.

[95] Al-Nabolsi A., Faizee F., Smith Z., Dixit A., Kaplan P.V., Heal K., Shatila M. (2025). A rare manifestation of human immunodeficiency virus masquerading as refractory cardiogenic shock. Am. J. Respir. Crit. Care Med..

[96] Ali S.H., Seghers M., Pham H., Khan U.N., Trivedi L., Sundaram K., Cauthen C. (2025). Fulminant myocarditis in the setting of acute HIV and group A Streptococcus pharyngitis. J. Am. Coll. Cardiol..

[97] Andrews T.Q., McBride P., Abdelhai O.S., Toiv A., Zimmerman A., Cowger J.A. (2025). Cardiogenic shock secondary to human immunodeficiency virus induced myocarditis. J. Am. Coll. Cardiol..

[98] Fatmi O., Anderson J., Bowler M., Patel R. (2025). Pericardial effusion and renal failure as presenting symptoms of HIV. Crit. Care Med..

[99] Mittal A., Alalwan Y., Cowger J. (2025). The ART of HAART: Rapid recovery of HIV associated cardiomyopathy. J. Heart Lung Transplant..

